# The association between domain-specific physical activity in adults and Parkinson’s disease and all-cause mortality: a NHANES study from 2007 to 2018

**DOI:** 10.5114/biolsport.2026.158669

**Published:** 2026-01-23

**Authors:** Wenwen Diao, Shunxiong Tang

**Affiliations:** 1 Department of Physical Education, Kookmin University, Seoul ‘02707, South Korea; 2 Interventional Department, Affiliated Zhongshan Hospital of Dalian University, Dalian 116001, China

**Keywords:** Domain-specific physical activity, Mortality, Parkinson’s disease, National Health and Nutrition, Examination Survey

## Abstract

This study aimed to examine the associations between physical activity (PA) of different domains and intensities with the risk of Parkinson’s disease (PD) and all-cause mortality among adults aged 40 years and older. A total of 13,960 participants from the National Health and Nutrition Examination Survey (NHANES) 2007–2018 were included. Multivariable logistic regression models were used to evaluate the associations between PA and PD prevalence. Kaplan–Meier survival curves with log-rank tests were applied to compare mortality across PA categories, and Cox proportional hazards models were employed to assess the joint effects of PA and PD on all-cause mortality. Sex-stratified subgroup analyses and multiple sensitivity analyses were also performed to confirm the robustness of the findings. After adjustment for potential confounders, vigorous occupational PA (OPA) (OR = 0.349, 95% CI: 0.181–0.674) and total vigorous PA (OR = 0.471, 95% CI: 0.260–0.853) were inversely associated with PD prevalence. Survival analyses demonstrated that higher levels of PA, regardless of domain or intensity, were significantly associated with lower mortality. Consistent results were observed in Cox regression models, where adherence to moderate or vigorous PA was associated with a 29.5% (HR = 0.705, 95% CI: 0.638–0.7781) and 29.0% (HR = 0.710, 95% CI: 0.610–0.826) reduction in mortality risk, respectively. Among participants with PD, those not engaging in vigorous OPA (HR = 1.483, 95% CI: 1.134–1.940) or total vigorous PA (HR = 1.504, 95% CI: 1.150–1.968) had significantly higher mortality than non-PD individuals, whereas those who were physically active exhibited mortality risks comparable to their non-PD counterparts. Sensitivity analyses yielded consistent results, supporting the robustness of these associations. Vigorous occupational and total vigorous physical activity were inversely associated with both PD prevalence and all-cause mortality among adults aged ≥ 40 years. Moreover, adherence to vigorous PA among individuals with PD may mitigate the excess mortality risk associated with the disease. Nevertheless, prospective cohort studies are warranted to confirm these findings and clarify the underlying mechanisms.

## INTRODUCTION

Parkinson’s disease (PD) is a common neurodegenerative disorder characterized by muscle rigidity, resting tremor, and bradykinesia. According to the World Health Organization (WHO, 2023), the global disability-adjusted life years (DALYs) attributable to PD have increased by 81% since 2000, while PD-related deaths have more than doubled, making it the fastest-growing neurodegenerative disease worldwide in terms of both prevalence and mortality [1]. Although pharmacological treatments such as levodopa can effectively relieve motor symptoms, no existing therapy has been shown to halt or reverse disease progression, largely due to the incomplete understanding of PD pathogenesis [2]. Given the chronic and progressive nature of PD, optimizing long-term outcomes and reducing the associated healthcare and caregiving burden have become urgent clinical priorities.

Physical activity (PA), defined by the WHO as any bodily movement produced by skeletal muscles that requires energy expenditure, serves as an important non-pharmacological intervention. Evidence suggests that strength training, running, and dancing can improve muscle strength, maintain postural stability, and reduce fall risk, thereby enhancing motor function in individuals with PD [3–5]. Beyond motor symptoms, PD is characterized by a broad range of nonmotor manifestations, including cognitive impairment, depression, anxiety, and fatigue [6]. The Parkinson’s Foundation Working Group has emphasized that PA not only improves physical performance but also confers psychological and cognitive benefits [7]. For instance, yoga, Tai Chi, and Qigong have been shown to alleviate depressive symptoms, enhance sleep quality, and improve overall quality of life in patients with PD [8, 9]. From a neuroprotective standpoint, exercise may mitigate lipopolysaccharide-induced inflammation in the dentate gyrus by suppressing astrocytic overactivation and reducing proinflammatory cytokine release, thereby attenuating cognitive decline [10]. Intervention studies have further demonstrated that engaging in approximately 60 minutes of light-to-moderate combined exercise daily significantly enhances global cognition and executive function in PD patients [11].

Domain-specific physical activity refers to the categorization of PA according to distinct life contexts or functional purposes, including leisure-time PA (LTPA), occupational PA (OPA), and transportrelated PA (TPA). Different PA domains may influence health outcomes through unique physiological and psychosocial mechanisms. However, most existing studies have concentrated on total PA or LTPA, leaving the roles of OPA and TPA less clearly defined and often debated. For example, some evidence indicates that LTPA is inversely associated with depression risk, whereas TPA and OPA show no significant associations [12]. In the context of PD, prior studies have reported that various forms of exercise can enhance cognitive function [13]. One investigation revealed sex-specific inverse associations between LTPA and OPA and PD risk [14], whereas another found no significant association between OPA and PD risk [15].

Building upon these findings, the present study aimed to explore the associations between domain-specific PA and both PD risk and all-cause mortality in adults aged 40 years and older. Specifically, it examined the effects of LTPA, OPA, and TPA on PD prevalence and mortality, using data from the National Health and Nutrition Examination Survey (NHANES) 2007–2018.

## MATERIALS AND METHODS

### Study population

**FIG. 1:**
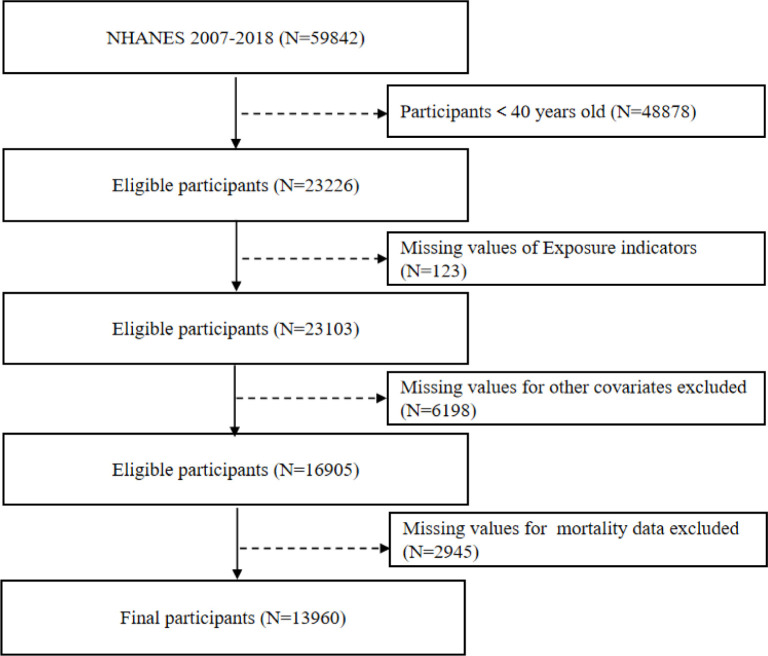
Flow chart of participants selection.

This study analyzed data from 59,842 participants enrolled in the NHANES between 2007 and 2018. Participants younger than 40 years (N = 48,878) were initially excluded. Subsequently, individuals with missing information on physical activity (N = 123) were removed, yielding 23,103 eligible participants. After further excluding participants with incomplete covariate data (N = 6,198) and those without mortality follow-up information (N = 2,945), a final analytic sample of 13,960 participants was retained for analysis (Figure 1).

### Assessment of PA

PA was assessed using the Global Physical Activity Questionnaire (GPAQ) and categorized into three domains: occupational PA (OPA), transport-related PA (TPA), and leisure-time PA (LTPA) [16]. For each domain, participants reported the frequency (days per week) and duration (minutes per day) of moderate- and/or vigorous-intensity activities during a typical week, from which weekly activity minutes were calculated.

According to the World Health Organization (WHO) recommendations, adults should engage in at least 150 minutes of moderateintensity or 75 minutes of vigorous-intensity aerobic activity per week. Based on this guideline, participants were classified into two groups: those meeting and those not meeting the recommended PA levels.

During the calculation process, the minutes of vigorous-intensity activity were weighted twice (i.e., vigorous minutes × 2 + moderate minutes) to derive the total activity volume for each domain (OPA total, LTPA total, and TPA total), as well as the overall physical activity level (Total PA). In addition, compliance with moderate-intensity (≥ 150 min/week) and vigorous-intensity (≥ 75 min/week) recommendations was calculated separately. Since TPA included only moderate-intensity activity, it was excluded from the vigorous-intensity compliance analysis. Details regarding the calculation of PA across domains are provided in Supplementary Table 1.

### Assessment of PD

In this study, the identification of PD cases was based on the “anti-Parkinson’s drug” entry recorded under the “secondary category name” field in the NHANES prescription medication files. This approach relies on participants’ self-reported use of prescription medications. It is important to note that this method is inherently limited by the scope of drug categories and coding available in the NHANES database and, therefore, can only identify individuals currently receiving pharmacological treatment for PD. In other words, participants were classified as PD patients if they reported taking anti-Parkinsonian medications, while those who did not report such use were classified as non-PD participants [17].

### Covariance

Covariates included in the analysis were sex, age, race, education level, marital status, smoking status, body mass index (BMI), family poverty income ratio (PIR), comorbidities (diabetes, hypertension, cardiovascular disease, and depression), and clinical laboratory parameters including alanine aminotransferase (ALT), aspartate aminotransferase (AST), blood urea nitrogen (BUN), and creatinine.

Participants who provided a positive response to the smoking question in the NHANES questionnaire were considered to have a history of smoking [18]. The presence of diabetes, hypertension, or cardiovascular disease was determined based on participants’ self-reports of being previously diagnosed by a physician. The PIR, which reflects household economic status, was dichotomized into two groups: < 2 and ≥ 2 [19]. BMI was categorized using a cutoff value of 25 kg/m^2^, with values ≥ 25 defined as overweight or obese.

Depressive symptoms were assessed using the Patient Health Questionnaire-9 (PHQ-9), a widely validated and reliable screening instrument developed based on DSM-IV diagnostic criteria for depressive disorders [20]. The PHQ-9 consists of nine items, with a total possible score ranging from 0 to 27. A total score ≥ 10 was considered indicative of major depressive disorder (MDD).

### Assessment of mortality

The mortality status of participants was determined using the National Death Index (NDI) maintained by the National Center for Health Statistics (NCHS). The NDI integrates multiple sources, including official death certificates, and covers all registered deaths across the United States. Mortality data were updated through December 31, 2019.

### Statistical analysis

The statistical analyses for this study incorporated sample weights, clustering, and stratification due to the complex multi-stage stratified probability survey design employed in NHANES. Continuous variables were presented as mean (standard deviation, SD), and differences between groups were evaluated using Student’s t-tests. Categorical variables were expressed as numbers (percentages) and compared using the chi-square test. To examine the association between PA and PD prevalence, logistic regression models were constructed with noncompliance to PA recommendations as the reference group. Odds ratios (ORs) and 95% confidence intervals (CIs) were reported for each PA domain and intensity category. Survival analyses were conducted using rank-sum tests and Kaplan–Meier curves, followed by Cox proportional hazards regression to evaluate the joint effects of PA and PD on all-cause mortality. Three hierarchical models were employed: Unadjusted Model: no covariate adjustment. Model 1: adjusted for sex, age, race, BMI, education level, marital status, and PIR. Model 2: further adjusted for hypertension, diabetes, cardiovascular disease, smoking status, AST, ALT, BUN, creatinine, and depressive symptoms, based on Model 1. Sex-stratified subgroup analyses were performed to explore potential effect modification. Covariates included in the Cox models were identical to those used in the logistic regression analyses. Hazard ratios (HRs) and 95% CIs were reported for all survival analyses. All statistical analyses were conducted using R software (version 4.1.1), with a two-sided p-value < 0.05 considered statistically significant.

### Sensitivity analyses

To verify the robustness of our findings, three sensitivity analyses were conducted.

First, to assess the potential influence of dietary quality on the associations between physical activity, PD prevalence, and all-cause mortality, we further adjusted for dietary intake of total energy, protein, carbohydrates, and total fat on the basis of Model 2 [21].

Second, given the cross-sectional nature of NHANES and the diagnostic challenges in differentiating early-stage idiopathic PD (G20) from rapidly progressive atypical parkinsonian syndromes (e.g., progressive supranuclear palsy and multiple system atrophy), there was a potential risk of misclassification bias. To evaluate its impact, and build upon Sensitivity Analysis 1, we excluded participants who died within two years after baseline to minimize potential reverse causation, and reanalyzed the associations between PA, PD prevalence, and all-cause mortality [22].

Finally, based on the second sensitivity analysis, self-reported health status and mobility limitations were further incorporated as additional covariates to control for potential residual confounding [23].

## RESULTS

### Baseline characteristics of selected participants

Baseline characteristics of the 13,960 included participants were compared according to PD status (Table 1). Compared with the non-PD group, individuals with PD had a lower overall PA compliance rate (49.85% vs. 60.82%), particularly for vigorous-intensity activity (14.18% vs. 29.82%). Demographically, PD participants were older and had a higher proportion of non-Hispanic White individuals. The prevalence of hypertension, cardiovascular disease, and depression was significantly higher in the PD group, and a greater proportion had low income (PIR < 2). Regarding lifestyle and health status, PD participants were more likely to report poor overall health, severe mobility limitations, and lower dietary protein intake, suggesting a less favorable functional and nutritional profile compared with non-PD individuals. Additionally, ALT levels were significantly lower in the PD group.

**TABLE 1:** Baseline characteristics of adult participants according to PD in NHANES 2007–2018

Parkinson Disease				

Characteristic	Overall, N = 13960	No, N = 13760	Yes, N = 200	P Value
Total PA compliance, n (%)	7640 (60.66%)	7562 (60.82%)	78 (49.85%)	0.039

OPA compliance, n (%)	4429 (36.07%)	4384 (36.20%)	45 (26.99%)	0.113

LTPA compliance, n (%)	3832 (32.99%)	3798 (33.11%)	34 (24.71%)	0.091

TPA compliance, n (%)	1652 (10.55%)	1636 (10.59%)	16 (8.10%)	0.386

Total moderate compliance, n (%)	6637 (52.47%)	6567 (52.57%)	70 (45.27%)	0.171

OPA moderate compliance, n (%)	3772 (31.54%)	3733 (31.64%)	39 (24.57%)	0.221

LTPA moderate compliance, n (%)	2690 (22.33%)	2662 (22.38%)	28 (18.78%)	0.365

Total vigorous compliance, n (%)	3424 (29.60%)	3401 (29.82%)	23 (14.18%)	0.001

OPA vigorous compliance, n (%)	2121 (17.13%)	2107 (17.29%)	14 (6.09%)	< 0.001

LTPA vigorous compliance, n (%)	1644 (15.34%)	1635 (15.44%)	9 (8.10%)	0.101

Sex, n (%)				0.144

Female	7041 (52.09%)	6940 (52.00%)	101 (58.74%)	

Male	6919 (47.91%)	6820 (48.01%)	99 (41.26%)	

Age (years)	57.66 ± 11.50	57.61 ± 11.47	60.81 ± 12.58	0.031

BMI, n (%)				0.456

< 25 kg/m^2^	3313 (24.69%)	3267 (24.64%)	46 (27.97%)	

≥ 25 kg/m^2^	10647 (75.31%)	10493 (75.36%)	154 (72.03%)	

Race, n (%)				< 0.001

Other Race	2788 (10.89%)	2764 (10.97%)	24 (5.28%)	

Non-Hispanic White	6338 (73.85%)	6206 (73.69%)	132 (84.99%)	

Mexican American	1947 (6.00%)	1930 (6.04%)	17 (3.20%)	

Non-Hispanic Black	2887 (9.26%)	2860 (9.30%)	27 (6.53%)	

Diabetes, n (%)	2592 (13.94%)	2541 (13.87%)	51 (18.79%)	0.109

Hypertension, n (%)	6719 (42.77%)	6602 (42.64%)	117 (52.06%)	0.031

Smoke, n (%)	6800 (47.89%)	6694 (47.89%)	106 (47.94%)	0.992

Education status, n (%)				0.511

above high school	7103 (60.79%)	7007 (60.87%)	96 (55.28%)	

high school	3260 (23.90%)	3214 (23.87%)	46 (26.45%)	

under high school	3597 (15.31%)	3539 (15.27%)	58 (18.26%)	

Marital status, n (%)				0.614

Married/Living with Partner	8693 (68.30%)	8575 (68.32%)	118 (66.61%)	

Never married	1106 (6.84%)	1080 (6.81%)	26 (9.02%)	

Widowed/Divorced/Separated	4161 (24.86%)	4105 (24.87%)	56 (24.37%)	

Cardiovascular disease, n (%)	2164 (12.44%)	2093 (12.24%)	71 (25.93%)	< 0.001

PIR, n (%)				0.031

< 2	6385 (29.43%)	6273 (29.291%)	112 (38.97%)	

≥ 2	7575 (70.57%)	7487 (70.71%)	88 (61.04%)	

AST (U/L)	25.59 ± 15.37	25.60 ± 15.43	24.94 ± 9.82	0.925

ALT (U/L)	24.93 ± 19.40	24.96 ± 19.48	22.76 ± 12.21	0.034

BUN (mg/dL)	14.84 ± 5.71	14.82 ± 5.69	16.20 ± 7.10	0.081

Creatinine (mg/dL)	0.91 ± 0.36	0.91 ± 0.36	0.93 ± 0.24	0.085

Depression, n (%)	1257 (7.75%)	1218 (7.59%)	39 (18.81%)	< 0.001

Severe health status, n (%)	604.00 (2.87%)	579.00 (2.78%)	25.00 (9.22%)	< 0.001

Severe mobility limit, n (%)	782.00 (4.42%)	759.00 (4.32%)	23.00 (11.35%)	< 0.001

Energy, kcal/day	2,055.02 ± 791.42	2,055.84 ± 792.61	1,997.78 ± 702.36	0.428

Protein, g/day	80.50 ± 33.78	80.61 ± 33.83	72.67 ± 29.01	0.003

**Total fat, g/day**	81.17 ± 38.63	81.22 ± 38.67	77.94 ± 35.48	0.370

**Carbohydrate, g/day**	241.66 ± 101.11	241.56 ± 101.15	248.57 ± 98.03	0.516

BMI, body mass index; PIR, poverty income ratio; AST, aspartate aminotransferase; ALT, alanine aminotransferase; BUN,blood urea nitrogen.

Supplementary Table 2 presents baseline characteristics stratified by all-cause mortality status. Among the 13,960 participants, a total of 1,860 deaths were recorded during follow-up. Compared with survivors, individuals in the death group were older (P < 0.001), more likely to be male (P < 0.001), and had a higher prevalence of diabetes, hypertension, cardiovascular disease, and depression (all P < 0.001). Significant differences were also observed in BMI, race, marital status, PIR, education level, smoking status, and physical activity levels, as well as in biochemical indicators related to liver and kidney function (AST, ALT, BUN, and creatinine) (all P < 0.05). In addition, participants in the death group were more likely to report poor overall health and severe mobility limitations, and had lower dietary intake of energy, protein, total fat, and carbohydrates compared with survivors, indicating poorer functional and nutritional status among those who died.

### Association between PA and PD

Next, we evaluated the association between compliance in different PA domains and PD risk through logistic regression analysis (Table 2). The results showed that there was no significant association between overall PA, OPA, TPA, and LTPA compliance and PD risk. Although in the univariate model without adjusting for any confounding factors, the overall risk of PD was lower among PAeligible individuals (P = 0.040), this association no longer had statistical significance after gradually adjusting for potential confounding factors (All P > 0.05).

**TABLE 2:** Logistic regression analysis to identify the association between domain-specific PA and PD.

	Unadjusted Model		Model 1		Model 2	

	OR (95% CI)	P value	OR (95% CI)	P value	OR (95% CI)	P value
**Total PA compliance**						
No	1 (Ref)		1 (Ref)		1 (Ref)	
Yes	0.640 (0.418–0.980)	0.040	0.714 (0.462–1.102)	0.126	0.771 (0.490–1.214)	0.257

**OPA compliance**						
No	1 (Ref)		1 (Ref)		1 (Ref)	
Yes	0.651 (0.381–1.113)	0.115	0.683 (0.401–1.162)	0.157	0.695 (0.411–1.176)	0.172

**TPA compliance**						
No	1 (Ref)		1 (Ref)		1 (Ref)	
Yes	0.744 (0.378–1.465)	0.388	0.771 (0.408–1.457)	0.418	0.861 (0.448–1.653)	0.647

**LTPA compliance**						
No	1 (Ref)		1 (Ref)		1 (Ref)	
Yes	0.663 (0.410–1.073)	0.093	0.744 (0.431–1.286)	0.285	0.805 (0.453–1.433)	0.455

Model 1: Adjusted for sex, age, race, BMI, education level, marital status, and PIR.

Model 2: Further adjusted for hypertension, diabetes, cardiovascular disease, smoking status, AST, ALT, BUN, creatinine, and depression, based on Model 1.

The logistic regression analysis assessing the association between different intensities of PA and PD risk (Table 3) revealed a significant inverse association between overall vigorous-intensity PA and PD risk, which remained significant after full adjustment for potential confounders (P = 0.014). Among PA domains, vigorous OPA exhibited the most pronounced protective effect against PD (P = 0.002), whereas no significant associations were observed for vigorous or moderate LTPA. In contrast, moderate-intensity PA, whether overall or domain-specific, was not significantly associated with PD risk across any model.

**TABLE 3:** Logistic regression analysis to identify the association between different intensities of domain-specific PA and PD

	Unadjusted Model		Model 1		Model 2	

	OR (95% CI)	P value	OR(95% CI)	P value	OR (95% CI)	P value
**Total moderate compliance**						
No	1 (Ref)		1 (Ref)		1 (Ref)	
Yes	0.746 (0.489–1.139)	0.172	0.798 (0.523–1.216)	0.288	0.854 (0.554–1.318)	0.470

**Total vigorous compliance**						
No	1 (Ref)		1 (Ref)		1 (Ref)	
Yes	0.389 (0.217–0.697)	0.002	0.449 (0.250–0.806)	0.008	0.471 (0.260–0.853)	0.014

**OPA moderate compliance**						
No	1 (Ref)		1 (Ref)		1 (Ref)	
Yes	0.704 (0.398–1.245)	0.224	0.733 (0.419–1.283)	0.272	0.740 (0.425–1.287)	0.280

**OPA vigorous compliance**						
No	1 (Ref)		1 (Ref)		1 (Ref)	
Yes	0.310 (0.161–0.597)	< 0.001	0.340 (0.177–0.651)	0.001	0.349 (0.181–0.674)	0.002

**LTPA moderate compliance**						
No	1 (Ref)		1 (Ref)		1 (Ref)	
Yes	0.802 (0.494–1.301)	0.366	0.839 (0.493–1.426)	0.511	0.902 (0.520–1.562)	0.708

**LTPA vigorous compliance**						
No	1 (Ref)		1 (Ref)		1 (Ref)	
Yes	0.482 (0.197–1.179)	0.108	0.594 (0.241–1.462)	0.252	0.628 (0.252–1.565)	0.312

Model 1: Adjusted for sex, age, race, BMI, education level, marital status, and PIR.

Model 2: Further adjusted for hypertension, diabetes, cardiovascular disease, smoking status, AST, ALT, BUN, creatinine, and depression, based on Model 1.

These findings suggest that only vigorous-intensity OPA may confer a protective effect in reducing the risk of PD, while moderate-intensity PA appears unrelated to PD risk.

### Association between PA and all-cause mortality

**FIG. 2:**
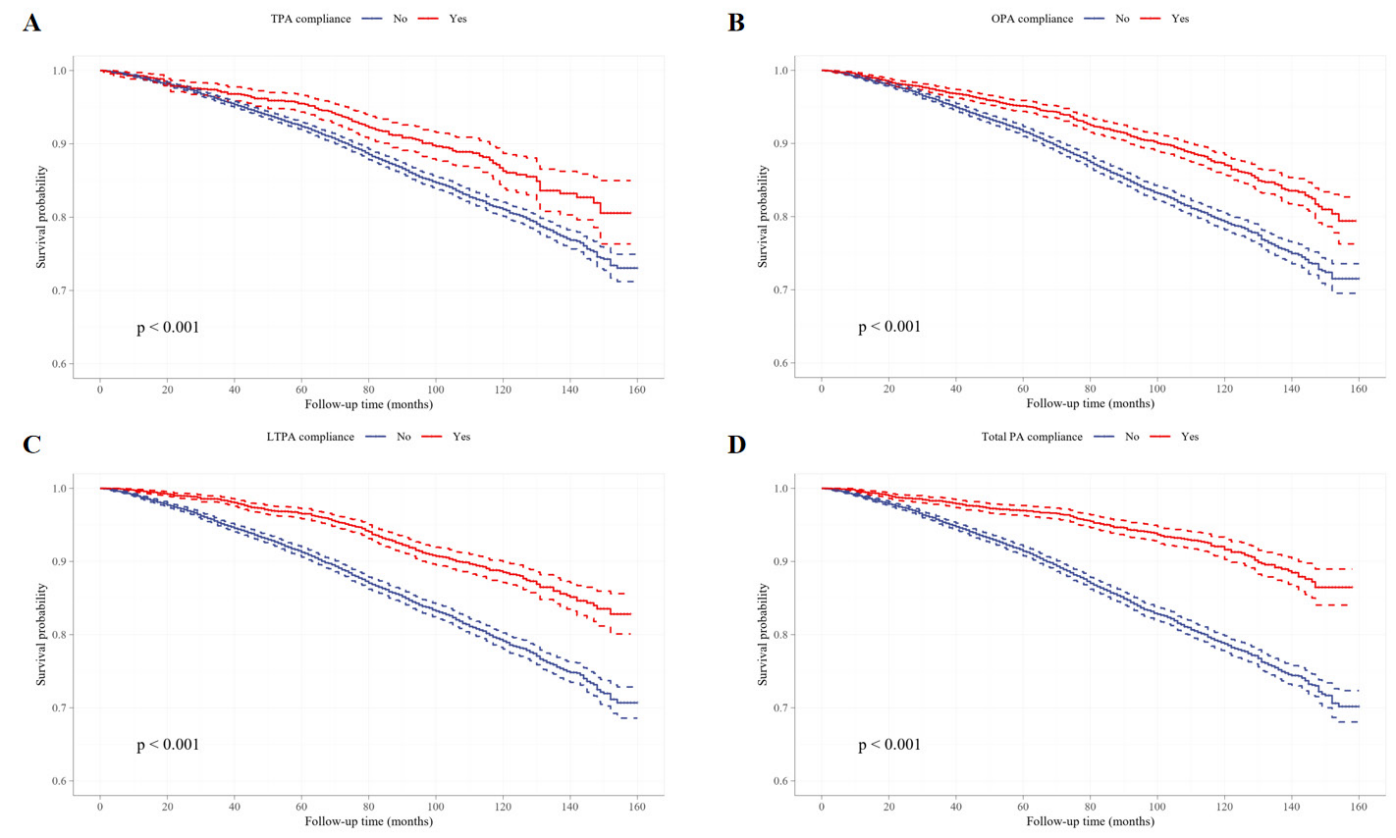
The survival curves of domain-specific PA and non-PA participants. Kaplan-Meier survival curves of TPA compliance (A), OPA compliance (B), LTPA compliance (C) and total PA compliance (D).

**FIG. 3:**
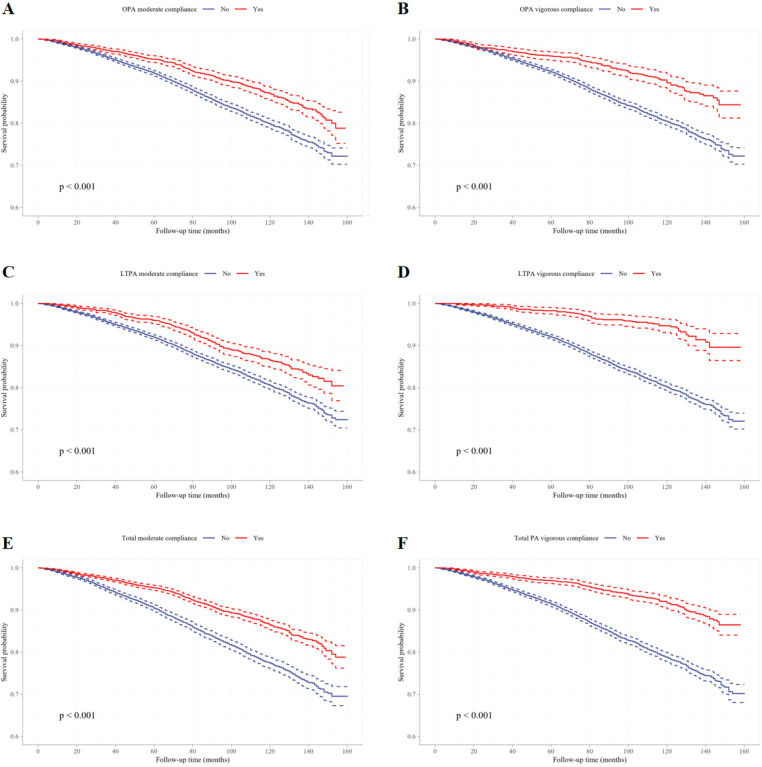
The survival curves for different intensity of domain-specific PA and non-PA participants. Kaplan-Meier survival curves of moderate OPA compliance (A), vigorous OPA compliance (B), moderate LTPA compliance (C), vigorous LTPA compliance (D), moderate total PA compliance (E), and vigorous total PA compliance (F).

To further investigate the relationship between PA and all-cause mortality, Kaplan–Meier survival curves were plotted and compared using the Log-rank test (Figure 2, Figure 3). In all PA domains and intensity categories, participants meeting the PA recommendations exhibited significantly higher survival probabilities than those who did not (all P < 0.0001), indicating a robust negative association between PA engagement and mortality risk.

The Cox regression analysis (Table 4) further substantiated these findings. Across total PA and its domains (OPA, TPA, and LTPA), compliance with PA guidelines was consistently associated with a significantly lower risk of all-cause mortality. In the fully adjusted model, individuals meeting the overall PA criteria had a 29.6% lower risk of death compared to non-compliant individuals (P < 0.001). Domain-specific analyses demonstrated that meeting OPA, TPA, or LTPA recommendations similarly conferred significant protective total PA compliance (E), and vigorous total PA compliance (F). effects (all P < 0.05). These results highlight PA compliance as a strong and independent protective factor for mortality risk.

**TABLE 4:** Cox regression analysis of the association between specific domain-specific PA and adult mortality.

	Unadjusted Model		Model 1		Model 2	

	HR (95% CI)	P value	HR (95% CI)	P value	HR (95% CI)	P value
**Total PA compliance**						
No	1 (Ref)		1 (Ref)		1 (Ref)	
Yes	0.508 (0.463, 0.557)	< 0.001	0.641 (0.582, 0.707)	< 0.001	0.704 (0.638, 0.776)	< 0.001

**OPA compliance**						
No	1 (Ref)		1 (Ref)		1 (Ref)	
Yes	0.605 (0.542, 0.674)	< 0.001	0.755 (0.675, 0.845)	< 0.001	0.817 (0.73, 0.915)	< 0.001

**TPA compliance**						
No	1 (Ref)		1 (Ref)		1 (Ref)	
Yes	0.662 (0.564, 0.777)	< 0.001	0.738 (0.627, 0.867)	< 0.001	0.808 (0.686, 0.95)	0.010

**LTPA compliance**						
No	1 (Ref)		1 (Ref)		1 (Ref)	
Yes	0.521 (0.461, 0.588)	< 0.001	0.62 (0.547, 0.702)	< 0.001	0.656 (0.578, 0.744)	< 0.001

Model 1: Adjusted for sex, age, race, BMI, education level, marital status, and PIR.

Model 2: Further adjusted for hypertension, diabetes, cardiovascular disease, smoking status, AST, ALT, BUN, creatinine, and depression, based on Model 1.

Subsequent analyses stratified by PA intensity (Table 5) confirmed this consistent protective association. In the fully adjusted model, individuals meeting the moderate-intensity PA standard exhibited a 29.5% reduction in mortality risk (HR = 0.705, 95% CI: 0.638–0.778, P < 0.001), while those meeting the vigorous-intensity standard had a 29.0% reduction (HR = 0.710, 95% CI: 0.610–0.826, P < 0.001). From a domain-specific perspective, both OPA and LTPA demonstrated significant mortality risk reductions at either moderate or vigorous intensity levels.

**TABLE 5:** Cox regression analysis of the association between different intensities of domain-specific PA and mortality.

	Unadjusted Model		Model 1		Model 2	

	HR (95% CI)	P value	HR (95% CI)	P value	HR (95% CI)	P value
**Total moderate compliance**						
No	1 (Ref)		1 (Ref)		1 (Ref)	
Yes	0.566 (0.514, 0.622)	< 0.001	0.65 (0.589, 0.717)	< 0.001	0.705 (0.638, 0.778)	< 0.001

**Total vigorous compliance**						
No	1 (Ref)		1 (Ref)		1 (Ref)	
Yes	0.379 (0.327, 0.438)	< 0.001	0.635 (0.546, 0.739)	< 0.001	0.710 (0.610, 0.826)	< 0.001

**OPA moderate compliance**						
No	1 (Ref)		1 (Ref)		1 (Ref)	
Yes	0.618 (0.550, 0.694)	< 0.001	0.762 (0.677, 0.858)	< 0.001	0.821 (0.728, 0.924)	0.001

**OPA vigorous compliance**						
No	1 (Ref)		1 (Ref)		1 (Ref)	
Yes	0.508 (0.431, 0.599)	< 0.001	0.714 (0.603, 0.845)	< 0.001	0.784 (0.662, 0.929)	0.005

**LTPA moderate compliance**						
No	1 (Ref)		1 (Ref)		1 (Ref)	
Yes	0.653 (0.573, 0.745)	< 0.001	0.633 (0.554, 0.724)	< 0.001	0.655 (0.572, 0.749)	< 0.001

**LTPA vigorous compliance**						
No	1 (Ref)		1 (Ref)		1 (Ref)	
Yes	0.293 (0.230, 0.372)	< 0.001	0.568 (0.444, 0.726)	< 0.001	0.658 (0.514, 0.842)	< 0.001

Model 1: Adjusted for sex, age, race, BMI, education level, marital status, and PIR.

Model 2: Further adjusted for hypertension, diabetes, cardiovascular disease, smoking status, AST, ALT, BUN, creatinine, and depression, based on Model 1.

In summary, regardless of domain or intensity, compliance with PA recommendations was significantly and inversely associated with all-cause mortality risk, underscoring the vital role of both occupational and leisure-time PA in promoting long-term survival.

### The correlation between PA, PD, and mortality

To further investigate the potential protective effect of PA on the health of middle-aged and older adults, we focused on vigorous-intensity OPA compliance and overall vigorous PA compliance as key indicators. These two measures had shown significant associations with outcomes in previous logistic and Cox regression analyses (P < 0.05), suggesting a potential protective role for vigorous PA. Using these indicators, we examined the combined effects of PA and PD on all-cause mortality (Table 6).

**TABLE 6:** Cox regression analysis of PA and PD with all-cause mortality

Exposure	Group	Unadjusted Model		Model 1		Model 2	

		HR (95% CI)	P value	HR (95% CI)	P value	HR (95% CI)	P value
OPA vigorous compliance	No PD	1 (ref)		1 (ref)		1 (ref)	
	PD without OPA	2.519 (1.931, 3.287)	< 0.001	1.609 (1.231, 2.102)	< 0.001	1.483 (1.134, 1.940)	0.004
	PD with OPA	2.125 (0.531, 8.504)	0.287	1.407 (0.351, 5.637)	0.630	1.365 (0.340, 5.474)	0.661

Total vigorous compliance	No PD	1 (ref)		1 (ref)		1 (ref)	
	PD without total PA	2.670 (2.047, 3.484)	< 0.001	1.646 (1.259, 2.150)	< 0.001	1.504 (1.150, 1.968)	0.003
	PD with total PA	0.909 (0.227, 3.640)	0.893	0.914 (0.228, 3.662)	0.899	1.004 (0.250, 4.027)	0.995

Model 1: Adjusted for sex, age, race, BMI, education level, marital status, and PIR.

Model 2: Further adjusted for hypertension, diabetes, cardiovascular disease, smoking status, AST, ALT, BUN, creatinine, and depression, based on Model 1.

Note: Groups are defined by PD status and vigorous PA compliance. “No PD” indicates participants without PD. “PD without OPA/ total PA” indicates participants with PD who did not meet the corresponding vigorous PA guideline. “PD with OPA/total PA” indicates participants with PD who met the corresponding vigorous PA guideline.

In the vigorous OPA analysis, after adjusting for all covariates, PD patients who did not meet the OPA criteria showed a significantly higher risk of death compared with non-PD participants (P = 0.004). In contrast, among PD patients who met the vigorous OPA criteria, no significant difference in mortality risk was observed relative to the non-PD group (HR = 1.365, 95% CI: 0.340–5.474, P = 0.661). A similar pattern was seen in the analysis of overall vigorous PA: PD patients who did not meet the PA recommendations exhibited a significantly increased mortality risk (P = 0.003), whereas those who met the recommendations had a mortality risk comparable to non-PD participants (HR = 1.004, 95% CI: 0.250–4.027, P = 0.995). These findings suggest that engaging in vigorous OPA may help mitigate excess mortality risk among individuals with PD.

### Gender subgroup analysis

**FIG. 4:**
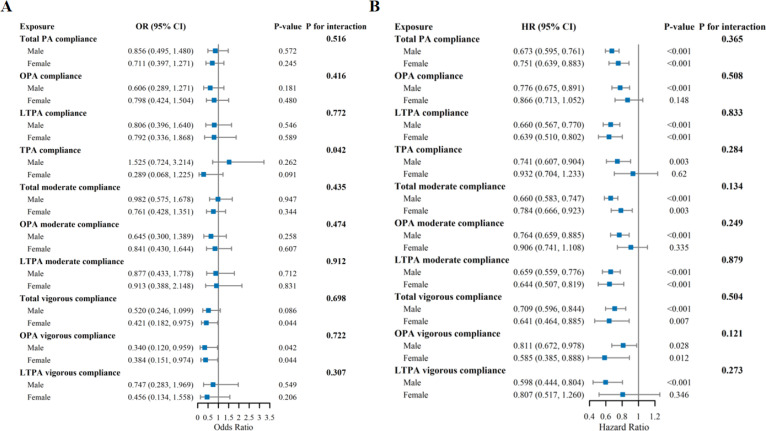
Gender-Stratified Associations Between Physical Activity Compliance and Parkinson’s Disease Prevalence and All-Cause Mortality. A: Logistic Regression Analysis for Parkinson’s Disease Prevalence; B Cox Regression Analysis for All-Cause Mortality.Adjusted for age, race, BMI, education status, marital status, PIR, hypertension, diabetes, cardiovascular disease, smoking status, AST, ALT, BUN, creatinine and depression.

Sex-stratified analyses (Figure 4) indicated that the protective associations of PA compliance with outcomes were generally consistent in both men and women, being associated with a reduced risk of PD and all-cause mortality.Although certain associations were not observed in women—for instance, OPA compliance, TPA compliance, moderate-intensity OPA compliance, and vigorous-intensity LTPA compliance with all-cause mortality—these associations were present in men. However, interaction tests were not statistically significant, suggesting that there were no meaningful sex differences in the overall protective effects of PA. One exception was observed for TPA compliance in relation to PD risk, which demonstrated a potential sex-specific effect (P for interaction = 0.042). Nonetheless, the overall pattern indicates that the beneficial effects of PA on PD and all-cause mortality are largely similar in men and women.

### Sensitivity analysis

After adjusting for dietary factors and further excluding participants who died within two years, as well as incorporating self-reported health status and activity limitations, compliance with vigorous OPA and total vigorous PA remained significantly associated with reduced risks in both logistic and Cox regression models. among PD patients who met vigorous PA recommendations, no significant excess mortality risk was observed compared to non-PD participants (Supplementary Tables 3–5).

## DISCUSSION

This study demonstrates that adherence to PA recommendations is associated with a reduced risk of both PD and all-cause mortality. Among different activity domains and intensities, vigorous total PA— particularly vigorous occupational PA—exhibited the strongest protective association with PD, and these relationships remained robust after adjustment for multiple potential confounders. Moreover, among individuals with PD, those who failed to meet the recommended levels of vigorous PA had a significantly higher risk of mortality compared with non-PD participants.

With respect to PD risk, OPA, broadly defined in this study, showed an inverse association with PD prevalence. This finding contrasts with earlier reports that conceptualized OPA more narrowly as highintensity manual labor and linked it to an increased PD risk. This discrepancy may primarily reflect differences in the operational definition of OPA. Previous studies have often described OPA as physically demanding, high-strain work requiring repetitive exertion or heavy lifting; for example, strenuous OPA involving repetitive tasks and heavy physical load has been associated with an elevated risk of myocardial infarction [24]. In contrast, our definition encompassed a broader spectrum of unpaid or low-intensity productive activities commonly undertaken by middle-aged and older adults, such as household chores, educational work, and farming. According to the U.S. Centers for Disease Control and Prevention, moderate- to highintensity household activities can contribute to the prevention of dementia, including Alzheimer’s disease [25]. Therefore, the association between OPA and PD appears to be highly contingent upon how OPA is defined. Future studies should incorporate objective and standardized measures of occupational activity intensity, volume, and frequency to clarify the complex relationship between work-related physical activity and neurological health [26].

No significant association was observed between LTPA and PD risk in this study, which may partly reflect the cross-sectional design. Previous evidence suggests that the protective effects of LTPA may occur only within specific temporal windows—either during early adulthood [27, 28] or several years before PD onset [29, 30]. The latter association could be biased by reverse causation, as prodromal symptoms of PD often lead to reduced activity levels. In contrast, studies tracking long-term PA trajectories have demonstrated that individuals maintaining high-intensity LTPA from young to middle adulthood exhibit the lowest PD risk [28, 30], consistent with several longitudinal findings. Collectively, these results suggest that the neuroprotective benefits of LTPA depend on its sustained engagement across the life course rather than short-term activity patterns [14]. Additionally, the relatively small number of PD cases with high LTPA exposure in our study may have limited the power to detect a significant association [31].

To explore sex-specific effects, stratified analyses revealed no overall sex differences. While some associations between PA and allcause mortality were observed in men, no significant relationships emerged in women across occupational, total, moderate occupational, or high-intensity leisure PA, and none of the interaction tests reached statistical significance. Prior studies have reported inconsistent findings: a Danish cohort found that high occupational activity increased mortality only in men, whereas another reported an inverse association exclusively in women [32, 33]. Such discrepancies likely arise from heterogeneity in population characteristics, PA classification, and confounding adjustment, suggesting that sex–PA interactions are context-dependent rather than biologically universal. Nevertheless, we observed potential sex heterogeneity in the association between total PA and PD risk (interaction P = 0.042), indicating possible sex differences in neuroprotective effects. Given the multiple testing involved, however, this result should be interpreted cautiously and regarded as hypothesis-generating for future mechanistic or prospective research investigating sex-specific biological pathways.

From a pathological perspective, various types of physical activity can effectively alleviate Parkinson’s disease symptoms through multi-faceted modulation of cytokine networks and related signaling pathways [34]. The core pathological features of PD include the loss of dopaminergic neurons in the substantia nigra pars compacta and abnormal aggregation of α-synuclein, often accompanied by neuroinflammation and dysregulation of neurotransmitters. Recent studies indicate that PA inhibits microglial ferroptosis via activation of the SLC7A11/ALOX12 axis, thereby enhancing phagocytic clearance of αsynuclein aggregates, reducing cerebral αsynuclein deposition, and significantly ameliorating neurologic deficits in PD mouse models [35]. Meanwhile, PA suppresses NLRP3 inflammasome activation and microglial overactivation through downregulation of the TLR4/MyD88/NFκB pathway, effectively attenuating neuronal damage in PD mice [36]. Furthermore, PA significantly upregulates the expression of bone morphogenetic protein and transforming growth factorβ2 [37, 38]. BMP6/BMP2 and TGFβ2 have been shown to promote dopaminergic synaptic development in nigrostriatal and mesolimbic neurons via Smad1/2 pathway activation, thereby improving neural signaling [39]. PA also improves non-motor symptoms in PD model rats by maintaining normal expression of RAGE in the frontal cortex and upregulating the neuroprotective PD-associated gene DJ1 [40]. The present study found that vigorous occupational PA significantly reduced PD risk, underscoring the dual therapeutic and preventive value of PA in PD patients. In contrast, moderate-intensity PA across various domains showed no significant association with PD risk. Previous research reported a positive dose–response relationship between exercise intensity and brain-derived neurotrophic factor levels: vigorous PA elevated BDNF by 30–50% in PD patients, whereas moderate-intensity PA raised it by only 10–15% [41]. BDNF enhances dopaminergic neuron survival and improves neurotransmission and motor performance in PD animal models by promoting neuroprotection and neuroregeneration [42]. These findings suggest that moderateintensity PA may not reach the threshold required for substantial neuroprotection, possibly explaining its limited role in modifying PD pathology compared to vigorous PA.

Previous studies have confirmed that regular exercise can reduce the risk of 25 or more non-communicable diseases and premature mortality among people of all ages [43]. Research has shown that for every 1 metabolic equivalent (MET) increase in physical activity, premature mortality decreases by 10–25% [44]. Specifically, PA can improve cardiac parasympathetic regulation and prevent ventricular fibrillation by reducing the sensitivity of β 2-adrenergic receptors, thereby improving arrhythmia [45]. At the same time, PA can increase the content of collagen and elastin in atherosclerotic plaque, reduce the volume of necrotic core, and reduce the burden of the whole plaque [46]. High intensity PA can also stimulate endothelial-dependent vasodilation by increasing the synthesis of nitric oxide, thereby regulating vascular tone and blood pressure, and reducing the risk of death in PD patients due to hypertension [47]. In addition, depression can have a negative impact on the interpersonal functioning and happiness of PD patients, which also leads to a higher mortality rate among PD patients. On the one hand, high-intensity PA can upregulate various neurotrophic factors and promote the regeneration and migration of hippocampal dentate gyrus neurons [48]. On the other hand, PA can improve patients’ depressive mood by activating serotonin type 3 receptors and promoting the synthesis and synaptic release of serotonin in the central nervous system [49].

### Limitations

This study has several limitations. First, PA was assessed using selfreported questionnaires rather than objective tools such as accelerometers, which could have provided more precise information on activity intensity, duration, and type. Moreover, PA was measured only at baseline, making it vulnerable to recall bias and unable to reflect temporal changes, which may have influenced the observed associations. Second, PD status was primarily identified through self-reported use of anti-PD medications without clinical validation or classification by disease phenotype. This approach may have reduced diagnostic accuracy and failed to capture heterogeneity in PA–outcome associations across disease stages. Although some misclassification of undiagnosed or untreated PD is possible, this definition has been widely used in previous NHANES-based analyses and remains the most practical given data constraints [50]. Third, despite adjusting for a broad set of known confounders, residual confounding from unmeasured factors—such as genetic susceptibility, environmental exposures, and occupational hazards—cannot be excluded. Future studies integrating genomic, environmental, and occupational data are warranted to address these potential sources of bias. Finally, the cross-sectional design of NHANES precludes causal inference regarding the relationships between PA, PD, and all-cause mortality. The absence of clinical indicators of PD severity (e.g., Hoehn–Yahr stage) also limited our ability to fully adjust for reverse causation arising from disease progression. To mitigate this bias, we adjusted for self-rated health, mobility limitations, and vital status; the consistency of results in sensitivity analyses supports the robustness of our findings. Nevertheless, longitudinal studies incorporating PD staging and objective PA monitoring are needed to better delineate causal pathways between PA and PD-related outcomes.

### Implications

Despite these limitations, the findings have important implications for public health and clinical practice. From a population health perspective, interventions should move beyond promoting leisure-time exercise to adopt an “all-domain” activity framework that integrates occupational, transport-related, and daily-living activities. Middleaged and older adults, in particular, should be encouraged to engage in moderate- to vigorous-intensity tasks—such as household chores, gardening, and active commuting—as feasible means of maintaining overall activity levels.

Clinically, high-intensity PA should be incorporated into comprehensive management plans for PD patients and at-risk individuals as a core therapeutic component to improve motor symptoms, mitigate comorbidities, and reduce mortality risk. Moreover, emerging evidence of sex-specific variations in total PA effects highlights the need for personalized activity recommendations in future clinical guidelines.

## CONCLUSIONS

This study demonstrates that physical activity of varying intensities can significantly reduce the risk of all-cause mortality among adults aged 40 years and older. In particular, vigorous OPA and total PA appear to exert protective effects against the prevalence of Parkinson’s disease. Future studies should integrate genomic and environmental data, adopt longitudinal designs, incorporate PD staging, and employ objective measures of physical activity to more comprehensively address residual confounding and elucidate the causal pathways linking PA with PD-related mortality.

## Supplementary Material

The association between domain-specific physical activity in adults and Parkinson’s disease and all-cause mortality: a NHANES study from 2007 to 2018

## Data Availability

The data and materials in the current study are available from the corresponding author on reasonable request.
