# Supplementary material for: The association between domain-specific physical activity in adults and Parkinson’s disease and all-cause mortality: a NHANES study from 2007 to 2018
Source: Biol Sport. 2026 Jan 23;43:795–813. doi: 10.5114/biolsport.2026.158669 (PMC13217145; doi:10.5114/biolsport.2026.158669)
Supplement: The association between domain-specific physical activity in adults and Parkinson’s disease and all-cause mortality: a NHANES study from 2007 to 2018 [file JBS-43-57443-s1.pdf]

SUPPLEMENTARY MATERIALS

SUPPLEMENTARY TABLE 1. Definition of domain-specific physical activity with varying intensities

Domain	Intensity Types	Calculation Method	Compliance Definition
Occupational PA	Moderate, Vigorous	Vigorous: days × minutes (≥ 75 min/session)<br>Moderate: days × minutes (≥ 150 min/session)<br>Weighted Score = Vigorous × 2 + Moderate	≥ 150 min/week
Transportation PA	Moderate only	Moderate: days × minutes	≥ 150 min/week
Leisure-Time PA	Moderate, Vigorous	Vigorous: days × minutes (≥ 75 min/session)<br>Moderate: days × minutes (≥ 150 min/session)<br>Weighted Score = Vigorous × 2 + Moderate	≥ 150 min/week
Total PA	Moderate, Vigorous	Total Score = Occupational Score + Leisure-Time Score + Transportation Score	≥ 150 min/week
Moderate-Intensity Compliance	Moderate only	Sum of all moderate-intensity minutes	≥ 150 min/week
Vigorous-Intensity Compliance	Vigorous only	Sum of all vigorous-intensity minutes	≥ 75 min/week

SUPPLEMENTARY TABLE 2. Baseline characteristics of adult participants according to all-cause mortality in NHANES 2007–2018.

Characteristic	All-cause mortality		P Value
	No, N = 12100	Yes, N = 1860	
Total PA compliance, n (%)	6911 (62.64%)	729 (41.53%)	< 0.001
OPA compliance, n (%)	4016 (37.26%)	413 (24.56%)	< 0.001
LTPA compliance, n (%)	3519 (34.48%)	313 (18.59%)	< 0.001
TPA compliance, n (%)	1488 (10.82%)	164 (8.03%)	0.010
Total moderate compliance, n (%)	5986 (54.02%)	651 (37.45%)	< 0.001
OPA moderate compliance, n (%)	3422 (32.65%)	350 (20.82%)	< 0.001
LTPA moderate compliance, n (%)	2433 (23.03%)	257 (15.56%)	< 0.001
Total vigorous compliance, n (%)	3221 (31.39%)	203 (12.29%)	< 0.001
OPA vigorous compliance, n (%)	1968 (17.94%)	153 (9.31%)	< 0.001
LTPA vigorous compliance, n (%)	1575 (16.49%)	69 (4.16%)	< 0.001
Sex, n (%)			< 0.001
Female	6295 (52.77%)	746 (45.50%)	
Male	5805 (47.23%)	1114 (54.50%)	
Age (years)	56.47 ± 10.87	69.10 ± 11.06	< 0.001
BMI, n (%)			0.002
< 25 kg/m^2	2771 (24.32%)	542 (28.21%)	
≥ 25 kg/m^2	9329 (75.68%)	1318 (71.79%)	
Race, n (%)			< 0.001
Other Race	2580 (11.33%)	208 (6.62%)	
Non-Hispanic White	5165 (73.15%)	1173 (80.62%)	
Mexican American	1814 (6.27%)	133 (3.37%)	
Non-Hispanic Black	2541 (9.25%)	346 (9.40%)	
Diabetes, n (%)	2068 (12.82%)	524 (24.75%)	< 0.001
Hypertension, n (%)	5518 (40.71%)	1201 (62.64%)	< 0.001
Smoke, n (%)	5649 (46.58%)	1151 (60.59%)	< 0.001
Education status, n (%)			< 0.001
Above high school	6392 (62.48%)	711 (44.48%)	
High school	2761 (23.45%)	499 (28.24%)	
Under high school	2947 (14.07%)	650 (27.28%)	
Marital status, n (%)			< 0.001
Married/Living with Partner	7742 (69.70%)	951 (54.75%)	
Never married	979 (6.88%)	127 (6.46%)	
Widowed/Divorced/Separated	3379 (23.42%)	782 (38.80%)	
Cardiovascular disease, n (%)	1511 (10.28%)	653 (33.33%)	< 0.001
PIR, n (%)			< 0.001
< 2	5310 (27.51%)	1075 (47.92%)	
≥ 2	6790 (72.49%)	785 (52.08%)	
AST (U/L)	25.34 ± 14.16	28.07 ± 24.00	< 0.001
ALT (U/L)	25.00 ± 16.19	24.23 ± 38.44	< 0.001
BUN (mg/dL)	14.54 ± 5.11	17.69 ± 9.28	< 0.001
Creatinine (mg/dL)	0.89 ± 0.30	1.09 ± 0.71	< 0.001
Depression, n (%)	1061 (7.49%)	196 (10.23%)	0.001
Severe health status, n (%)	435 (2.41%)	169 (7.31%)	< 0.001
Severe mobility limit, n (%)	550 (3.68%)	232 (11.59%)	< 0.001
Energy,kcal/day	2,075.34 ± 791.52	1,857.85 ± 763.02	< 0.001
Protein,g/day	81.38 ± 33.77	71.98 ± 32.68	< 0.001
**Total fat,g/day**	82.18 ± 38.77	71.39 ± 35.73	< 0.001
**Carbohydrate,g/day**	243.20 ± 101.51	226.67 ± 95.88	< 0.001

BMI, body mass index; PIR, poverty income ratio; AST, aspartate aminotransferase; ALT, alanine aminotransferase; BUN,blood urea nitrogen.

SUPPLEMENTARY TABLE 3. Sensitivity analysis of the relationship between domain-specific physical activity with varying intensities and PD.

Variable	Sensitivity Analysis 1 OR (95% CI)	Sensitivity Analysis 2 OR (95% CI)	Sensitivity Analysis 3 OR (95% CI)
**Total PA compliance**			
No		1 (ref)	
Yes	0.781 (0.493, 1.237)	0.766 (0.467, 1.256)	0.786 (0.480, 1.288)
**OPA compliance**			
No		1 (ref)	
Yes	0.688 (0.409, 1.156)	0.639 (0.361, 1.132)	0.642 (0.362, 1.139)
**LTPA compliance**			
No		1 (ref)	
Yes	0.827 (0.464, 1.475)	0.825 (0.449, 1.513)	0.853 (0.465, 1.565)
**TPA compliance**			
No		1 (ref)	
Yes	0.858 (0.443, 1.664)	0.876 (0.441, 1.739)	0.904 (0.456, 1.789)
**Total moderate compliance**			
No		1 (ref)	
Yes	0.854 (0.551, 1.323)	0.846 (0.530, 1.351)	0.871 (0.545, 1.391)
**OPA moderate compliance**			
No		1 (ref)	
Yes	0.732 (0.423, 1.266)	0.690 (0.380, 1.252)	0.696 (0.383, 1.262)
**LTPA moderate compliance**			
No		1 (ref)	
Yes	0.917 (0.531, 1.585)	0.899 (0.507, 1.595)	0.929 (0.525, 1.644)
**Total vigorous compliance**			
No		1 (ref)	
Yes	0.482 (0.263, 0.881)	0.488 (0.264, 0.899)	0.493 (0.268, 0.909)
**OPA vigorous compliance**			
No		1 (ref)	
Yes	0.346 (0.178, 0.672)	0.333 (0.167, 0.664)	0.335 (0.168, 0.671)
**LTPA vigorous compliance**			
No		1 (ref)	
Yes	0.664 (0.264, 1.667)	0.699 (0.278, 1.756)	0.712 (0.282, 1.796)

Sensitivity analysis 1 Adjusted for sex, age, race, BMI, education level, marital status, PIR, hypertension, diabetes, cardiovascular disease, smoking status, AST, ALT, BUN, creatinine, depression, and dietary intake of total energy, protein, carbohydrates, and total fat.

Sensitivity analysis 2 Further excluded participants who died within two years, with adjustments for sex, age, race, BMI, education level, marital status, PIR, hypertension, diabetes, cardiovascular disease, smoking status, AST, ALT, BUN, creatinine, depression, and dietary intake of total energy, protein, carbohydrates, and total fat.

Sensitivity analysis 3 Adjusted for sex, age, race, BMI, education level, marital status, PIR, hypertension, diabetes, cardiovascular disease, smoking status, AST, ALT, BUN, creatinine, depression, dietary intake of total energy, protein, carbohydrates, and total fat, as well as self-reported health status and activity limitations.

SUPPLEMENTARY TABLE 4. Sensitivity analysis of the relationship between domain-specific physical activity with varying intensities and all-cause mortality.

Variable	Sensitivity Analysis 1 HR (95% CI)	Sensitivity Analysis 2 HR (95% CI)	Sensitivity Analysis 3 HR (95% CI)
**Total PA compliance**			
No		1 (ref)	
Yes	0.699 (0.633, 0.772)	0.709 (0.594, 0.846)	0.722 (0.604, 0.862)
**OPA compliance**			
No		1 (ref)	
Yes	0.827 (0.738, 0.926)	0.645 (0.518, 0.803)	0.652 (0.524, 0.812)
**LTPA compliance**			
No		1 (ref)	
Yes	0.649 (0.571, 0.738)	0.695 (0.557, 0.868)	0.712 (0.570, 0.890)
**TPA compliance**			
No		1 (ref)	
Yes	0.793 (0.672, 0.937)	0.895 (0.680, 1.176)	0.897 (0.682, 1.181)
**Total moderate compliance**			
No		1 (ref)	
Yes	0.700 (0.633, 0.774)	0.728 (0.609, 0.869)	0.741 (0.620, 0.886)
**OPA moderate compliance**			
No		1 (ref)	
Yes	0.827 (0.733, 0.933)	0.641 (0.507, 0.810)	0.648 (0.512, 0.819)
**LTPA moderate compliance**			
No		1 (ref)	
Yes	0.649 (0.566, 0.745)	0.694 (0.549, 0.878)	0.713 (0.563, 0.903)
**Total vigorous compliance**			
No		1 (ref)	
Yes	0.718 (0.617, 0.837)	0.611 (0.460, 0.812)	0.616 (0.464, 0.819)
**OPA vigorous compliance**			
No		1 (ref)	
Yes	0.798 (0.673, 0.946)	0.648 (0.468, 0.896)	0.653 (0.472, 0.904)
**LTPA vigorous compliance**			
No		1 (ref)	
Yes	0.658 (0.512, 0.845)	0.649 (0.419, 1.005)	0.654 (0.422, 1.013)

Sensitivity analysis 1 Adjusted for sex, age, race, BMI, education level, marital status, PIR, hypertension, diabetes, cardiovascular disease, smoking status, AST, ALT, BUN, creatinine, depression, and dietary intake of total energy, protein, carbohydrates, and total fat.

Sensitivity analysis 2 Further excluded participants who died within two years, with adjustments for sex, age, race, BMI, education level, marital status, PIR, hypertension, diabetes, cardiovascular disease, smoking status, AST, ALT, BUN, creatinine, depression, and dietary intake of total energy, protein, carbohydrates, and total fat.

Sensitivity analysis 3 Adjusted for sex, age, race, BMI, education level, marital status, PIR, hypertension, diabetes, cardiovascular disease, smoking status, AST, ALT, BUN, creatinine, depression, dietary intake of total energy, protein, carbohydrates, and total fat, as well as self-reported health status and activity limitations.

SUPPLEMENTARY TABLE 5. Sensitivity analysis of the relationship between PA and PD with all-cause mortality.

Exposure	Group	Sensitivity Analysis 1 HR (95% CI)	Sensitivity Analysis 2 HR (95% CI)	Sensitivity Analysis 3 HR (95% CI)
OPA vigorous compliance	No PD		1 (Ref)	
	PD without OPA	1.435 (1.089, 1.892)	1.628 (1.035, 2.56)	1.649 (1.048, 2.592)
	PD with OPA	1.348 (0.336, 5.412)	1.600 (0.224, 11.435)	1.363 (0.190, 9.761)
Total vigorous compliance	No PD		1 (Ref)	
	PD without total PA	1.458 (1.106, 1.922)	1.664 (1.058, 2.619)	1.687 (1.073, 2.654)
	PD with total PA	0.975 (0.243, 3.913)	1.124 (0.157, 8.034)	0.994 (0.139, 7.112)

Sensitivity analysis 1 Adjusted for sex, age, race, BMI, education level, marital status, PIR, hypertension, diabetes, cardiovascular disease, smoking status, AST, ALT, BUN, creatinine, depression, and dietary intake of total energy, protein, carbohydrates, and total fat.
Sensitivity analysis 2 Further excluded participants who died within two years, with adjustments for sex, age, race, BMI, education level, marital status, PIR, hypertension, diabetes, cardiovascular disease, smoking status, AST, ALT, BUN, creatinine, depression, and dietary intake of total energy, protein, carbohydrates, and total fat.
Sensitivity analysis 3 Adjusted for sex, age, race, BMI, education level, marital status, PIR, hypertension, diabetes, cardiovascular disease, smoking status, AST, ALT, BUN, creatinine, depression, dietary intake of total energy, protein, carbohydrates, and total fat, as well as self-reported health status and activity limitations.

Articles published in the Biology of Sport are licensed under an open access Creative Commons CC BY 4.0 license.
